# Self‐Charging Persistent Mechanoluminescence with Mechanics Storage and Visualization Activities

**DOI:** 10.1002/advs.202203249

**Published:** 2022-08-17

**Authors:** Yongqing Bai, Xiuping Guo, Birong Tian, Yongmin Liang, Dengfeng Peng, Zhaofeng Wang

**Affiliations:** ^1^ State Key Laboratory of Solid Lubrication Lanzhou Institute of Chemical Physics Chinese Academy of Sciences Lanzhou 730000 China; ^2^ State Key Laboratory of Applied Organic Chemistry College of Chemistry and Chemical Engineering Lanzhou University Lanzhou Gansu 730000 China; ^3^ Center of Materials Science and Optoelectronics Engineering University of Chinese Academy of Sciences Beijing 100049 China; ^4^ Key Laboratory of Optoelectronic Devices and Systems of the Ministry of Education and Guangdong Province College of Physics and Optoelectronic Engineering Shenzhen University Shenzhen 518060 China

**Keywords:** mechanics storage, mechanics visualization, persistent mechanoluminescence, self‐charging materials

## Abstract

Persistent mechanoluminescence (ML) with long lifetime is highly required to break the limits of the transient emitting behavior under mechanics stimuli. However, the existing materials with persistent ML are completely trap‐controlled, and a pre‐irradiation is required, which severely hinders the practical applications. In this work, a novel type of ML, self‐charging persistent ML, is created by compositing the Sr_3_Al_2_O_5_Cl_2_:Dy^3+^ (SAOCD) powders into flexible polydimethylsiloxane (PDMS) matrix. With no need for any pre‐irradiation, the as‐fabricated SAOCD/PDMS elastomer could exhibit intense and persistent ML under mechanics stimuli directly, which greatly facilitates its applications in mechanics lighting, displaying, imaging, and visualization. By investigating the matrix effects as well as the thermoluminescence, cathodoluminescence, and triboelectricity properties, the interfacial triboelectrification‐induced electron bombardment processes are demonstrated to be responsible for the self‐charged energy in  SAOCD under mechanics stimuli. Based on the unique self‐charging processes, the SAOCD/PDMS further exhibits mechanics storage and visualized reading activities, which brings novel ideas and approaches to deal with the mechanics‐related problems in the fields of mechanical engineering, bioengineering, and artificial intelligence.

## Graphical abstract

Herein, a novel type of mechanoluminescence (ML), self‐charging persistent ML, is reported by compositing the Sr_3_Al_2_O_5_Cl_2_:Dy^3+^ powders into flexible polydimethylsiloxane matrix. In addition to facilitating the applications in mechanics displaying and visualization, the unique self‐charging processes endow the materials with mechanics storage and visualized reading activities, showing broad application prospects in mechanical engineering, bioengineering, and artificial intelligence.

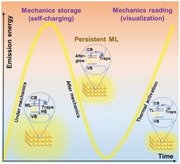

## 1 Introduction

Mechanoluminescence (ML) is a phenomenon featuring light‐emitting behaviors when they are subjected to mechanics stimuli, such as fracture, friction, compression, grinding and stretching.^[^ ^1^, ^2^ ^]^ ML directly builds a bridge between luminescence and mechanics, providing potential applications in passive lighting and displaying, high‐level information storage, artificial intelligent skin and wearable devices.^[^ ^3^, ^4^, ^5^, ^6^, ^7^, ^8^, ^9^, ^10^, ^11^ ^]^ In particular, by using the visible luminescent signals as the medium, ML could enable in situ, remote, distributed and visualized mechanics sensing for complex and irregular interfaces.^[^ ^12^, ^13^, ^14^, ^15^, ^16^, ^17^, ^18^, ^19^, ^20^, ^21^, ^22^, ^23^, ^24^ ^]^ Therefore, ML is highly promising to develop the next‐generation stress sensing and monitoring technology. Nevertheless, most of the current ML materials intrinsically exhibit instantaneous luminescence with a short lifetime ranging from nanoseconds (ns) to microseconds (ms).^[^ ^25^, ^26^ ^]^ Hence, when instantaneous ML is applied for mechanics displaying, imaging and visualization, the emitted ML signals can only be captured by the professional spectrometers, which is evidently inconvenient for the practical applications.

The development of long lifetime ML, i.e., persistent ML, is an effective approach to overcome the drawbacks derived from the instantaneous ML.^[^ ^27^ ^]^ In previous researches, very limited material systems have been reported to have persistent ML.^[^ ^28^, ^29^, ^30^, ^31^ ^]^ Smet and co‐workers reported that the BaSi_2_O_2_N_2_:Eu^2+^ can exhibit blue–green ML which remains visible after removing mechanics for several seconds.^[^ ^28^ ^]^ Our group further modified the persistent ML of BaSi_2_O_2_N_2_:Eu^2+^ by introducing Sr^2+^, and the developed Ba_0.5_Sr_0.5_Si_2_O_2_N_2_:Eu^2+^ exhibited an even higher ML intensity with the persistent time prolonged to tens of seconds.^[^ ^29^ ^]^ In addition, we also reported another two material systems, i.e., LiGa_5_O_8_^[^ ^30^ ^]^ and Sr_2_P_2_O_7_:Eu,Y,^[^ ^31^ ^]^ which showed green and blue persistent ML, respectively. The persistent light‐emitting feature after mechanics stimuli undoubtedly allows the displaying and visualization of mechanics to exist for a while, which is easy to be observed by the naked eyes or the common cameras. Therefore, it shows high convenience and practicability for mechanics visualization‐related applications. However, it should be noted that all of the reported persistent ML is actually established from the trap structure engineering, i.e., the pre‐filled energy in deep traps could be released to the shallow trap under mechanics stimuli, after which the energy in the shallow trap could be spontaneously and continuously transferred to the energy levels of luminescent centers producing the as‐observed persistent ML.^[^ ^29^, ^30^, ^31^ ^]^ Thus, the previous persistent ML always requires pre‐irradiation treatment on the materials to pre‐store energy, which increases the difficulty of the practical applications. As a result, it is highly desirable to develop self‐charging persistent ML that could self‐charge energy in the structure under mechanics stimuli to generate continuous ML with no need for pre‐irradiation.

The self‐charging persistent ML should be established on the self‐activating ML processes, in which the ML could be directly produced by the mechanics stimuli with no need for any other assistance or any pre‐stored energy. By introducing appropriate shallow tarp level and further connecting it to the ML self‐activating processes, it is feasible to realize the as‐expected self‐charging persistent ML. Very recently, we reported that the rare earth‐doped Sr_3_Al_2_O_5_Cl_2_ could exhibit self‐activating ML in the flexible polydimethylsiloxane (PDMS) matrix with the interfacial triboelectrification‐induced electron bombardment processes.^[^ ^32^ ^]^ Herein, by comprehensively considering the ML self‐activating processes as well as the shallow trap structure with spontaneous transfer activity, we synthesized Sr_3_Al_2_O_5_Cl_2_:Dy^3+^ (SAOCD) powders in this work, and investigated their ML performance by compositing with PDMS. It is found that the SAOCD powders could exhibit intense and self‐activating ML in PDMS under mechanics stimuli with no need for any pre‐irradiation. The most attractive feature is that the self‐activating ML of SAOCD/PDMS could last for a certain period, suggesting that we have successfully achieved the self‐charging persistent ML behaviors. In addition to facilitating the practical applications in mechanics displaying and visualization by the development of self‐charging persistent ML, the unique self‐charging processes further endow the SAOCD/PDMS composites with mechanics storage and visualized reading activities, which shows broad application prospects in the fields of mechanical engineering, bioengineering and artificial intelligence.

## 2 Results and Discussion

### 2.1 Synthesis and Characterizations of SAOCD Powders

**Figure 1:**
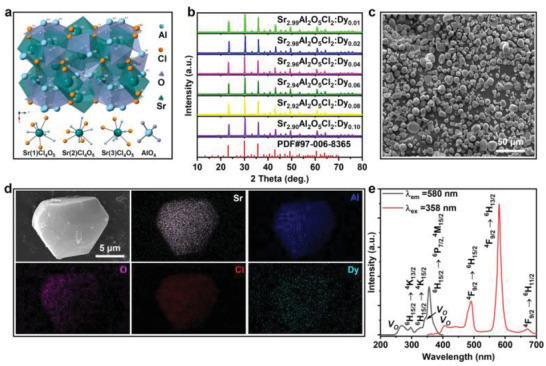
Structure and essential properties characterization of SAOCD. a) Crystal structure of the orthorhombic Sr_3_Al_2_O_5_Cl_2_. b) XRD patterns of the as‐synthesized SAOCD samples. c) SEM image of SAOCD. d) EDS mappings of SAOCD. e) PL and PLE spectra of SAOCD.

The crystal structure of Sr_3_Al_2_O_5_Cl_2_ is illustrated in **Figure** **1**a. According to the crystal data, Sr_3_Al_2_O_5_Cl_2_ belongs to the orthorhombic space group *P2_1_2_1_2_1_*, in which Al^3+^ and Sr^2+^ ions exist in the forms of [AlO_4_] tetrahedra and [SrO_5_Cl_4_] polyhedra, respectively.^[^ ^33^ ^]^ Considering the ionic radii of Sr^2+^ (R_CN = 9_ = 1.31 Å, where CN is the coordination number), Al^3+^ (R_CN = 4_ = 0.39 Å) and Dy^3+^ (R_CN = 9_ = 1.083 Å),^[^ ^34^ ^]^ the dopant of Dy^3+^ should substitute the ninefold coordination Sr^2+^ site in Sr_3_Al_2_O_5_Cl_2_ to form a solid solution, instead of the fourfold coordination Al^3+^ site. Figure 1b shows the X‐ray diffraction (XRD) patterns of the as‐synthesized SAOCD samples with different Dy^3+^ doping contents. It is found that the patterns are narrow and strong, suggesting that the samples have high crystallinity. Meanwhile, all the diffraction peaks match well with the standard inorganic crystal structure database PDF no. 97‐006‐8365 (ICSD file no. 68365), indicating that the single phase has been obtained for all samples and no any impurity phase is formed after doping with Dy^3+^ ions.

The scanning electron microscopy (SEM) image was measured to reveal the morphology and microstructure of SAOCD, as shown in Figure 1c. It is observed that the samples have sphere‐like morphology with a diameter of 5–20 µm. Based on the X‐ray energy dispersive spectrum (EDS) profiles (Figure S1, Supporting Information) and the elemental distribution mappings (Figure 1d), it can be confirmed that Sr, Al, O, Cl, and Dy have been uniformly distributed over the entire structure of the particles. Figure 1e shows the photoluminescence (PL) and photoluminescence excitation (PLE) spectra of the as‐synthesized SAOCD. It could be clearly observed that the excitation spectrum consists of five main excitation peaks when monitored at 580 nm, locating at 269, 296, 327, 343 and 358 nm, respectively. Among them, the excitation peaks at 296, 327, and 358 nm could be ascribed to the ^6^H_15/2_→^4^K_13/2_, ^6^H_15/2_→^4^K_15/2_, and ^6^H_15/2_→^6^P_7/2_, ^4^M_15/2_ transitions of Dy^3+^, respectively.^[^ ^35^, ^36^ ^]^ The peaks at 269 and 343 nm could be originated from the oxygen vacancy defects,^[^ ^37^ ^]^ which could be further confirmed by the atmosphere experiment (details are presented in Figure S2, Supporting Information). When excited by 358 nm, the SAOCD exhibits sharp emission peaks at 490, 580 and 674 nm, attributing to the characteristic ^4^F_9/2_→^6^H_15/2_, ^4^F_9/2_→^6^H_13/2_, ^4^F_9/2_→^6^H_11/2_ radiative transfers of Dy^3+^, respectively.^[^ ^36^ ^]^ It is also found in the PL emission spectra that there is a broad emission band from 400 to 450 nm produced by the oxygen vacancies.^[^ ^37^ ^]^ The above PL and PLE analyses provide us abundant electronic structure information of SAOCD, which should be helpful for investigating the ML performance.

### 2.2 Mechanoluminescence Performance of SAOCD/PDMS

**Figure 2:**
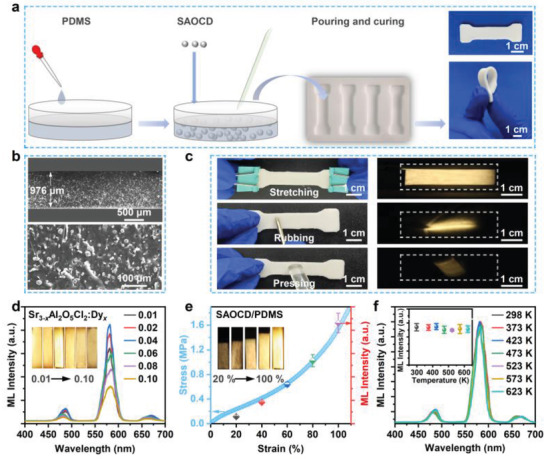
The preparation process and transient mechanoluminescence properties of SAOCD/PDMS composite elastomers. a) Schematic illustration of the fabrication process of SAOCD/PDMS composite elastomers. b) Cross‐sectional SEM images of the as‐fabricated SAOCD/PDMS composite elastomers under different magnifications. c) Optical and ML photographs of the as‐fabricated SAOCD/PDMS composite elastomers under stretching, rubbing, and pressing stimuli, respectively (effective sample size: 25 mm × 10 mm × 1 mm). d) ML spectra of the SAOCD/PDMS with various doping contents of Dy^3+^ ions (stretching mode, stretching frequency: 4 Hz, stretching strain: 100%). e) ML intensity and stress variations of the SAOCD/PDMS composite elastomers under different stretching strains (the error bars were obtained from three parallel tests). f) ML spectra of SAOCD/PDMS composite elastomers after been pre‐heat‐treated at various temperature for 10 min (the error bars were obtained from three parallel tests).

To facilitate the ML investigation, the SAOCD powders were composited with PDMS because of its efficient stress transfer ability and transparent characteristic. The fabrication processes are illustrated in **Figure** **2**a, and the as‐fabricated SAOCD/PDMS elastomer exhibits a dumbbell‐like shape with 25 mm in length and 10 mm in width. The cross‐sectional SEM images of the composite elastomer in Figure 2b suggest that SAOCD particles are evenly dispersed in the PDMS matrix, and the thickness of the elastomer is determined to be 976 µm. Figure S3 (Supporting Information) shows the stress‐strain curve of the SAOCD/PDMS composite elastomer tested on a universal testing machine, from which the elastic modulus and the tensile strength of the SAOCD/PDMS are obtained as 1.37 and 2.48 MPa, respectively. When mechanical actions are applied, such as stretching, rubbing and pressing, the SAOCD/PDMS composite elastomers exhibit intense light‐yellow ML as shown in Figure 2c, which could be easily captured by naked eyes. Figure 2d presents the ML spectra of SAOCD/PDMS elastomers with different doping contents of Dy^3+^ ions under the stretching mode (stretching frequency: 4 Hz, stretching strain: 100%). The samples all show light‐yellow ML composed of three main emission peaks at 490, 580, 674 nm, corresponding to the characteristic Dy^3+^ transitions of ^4^F_9/2_→^6^H_15/2_, ^4^F_9/2_→^6^H_13/2_, ^4^F_9/2_→^6^H_11/2_, respectively.^[^ ^36^ ^]^ No emissions from 400 to 450 nm can be found in the ML spectra, suggesting that the intrinsic oxygen vacancies are not involved in the ML processes. With the increase of Dy^3+^ content, the ML intensity of SAOCD/PDMS composite elastomers increases first and then decreases due to the concentration quenching,^[^ ^38^ ^]^ and the optimum doping content of Dy^3+^ is obtained as 0.04. The ML intensity also exhibits a direct responsiveness to the applied stress/strain. As shown in Figure 2e and Figure S4 (Supporting Information), with the increase of the applied strain from 20% to 100% (stress from 0.2 to 1.6 MPa), the ML intensity of the SAOCD/PDMS elastomer increases synchronously. Therefore, the ML of the composite elastomer can be utilized for the stretching stress/strain sensing. Particularly, the visualization and distributed characteristics of ML allow the elastomers to be used for the mechanics/force mapping,^[^ ^39^ ^]^ showing broad applications in the fields of structural health monitoring, bio‐mechanics monitoring and artificial intelligence.

It should be noted that the ML of the as‐fabricated SAOCD/PDMS elastomer can be directly generated with no need for any pre‐irradiation. This suggests that the ML of SAOCD/PDMS should be independent from the intrinsic traps, which is different from most of the previous researches.^[^ ^40^, ^41^ ^]^ To further confirm this viewpoint, the ML performance for the SAOCD/PDMS with various contents of traps was investigated. The samples were filled with energy by an ultraviolet (UV) lamp first, and then pre‐heat‐treated at various temperature for 10 min. In this case, the contents of the trapped carriers are gradually decreased with the increase of the pre‐treated temperature. When the pre‐treated temperature reaches 623 K, most of the carriers can be cleared as shown in Figure S5 (Supporting Information). However, it is attractive to find that although there are great changes on the contents of the trapped carriers in SAOCD/PDMS (Figure S5, Supporting Information), the ML intensity almost shows no any change as depicted in Figure 2f. This fully demonstrates that there should be a distinct ML process in SAOCD/PDMS that is independent on the trapped carriers.

### 2.3 Self‐Charging Persistent Mechanoluminescence

**Figure 3:**
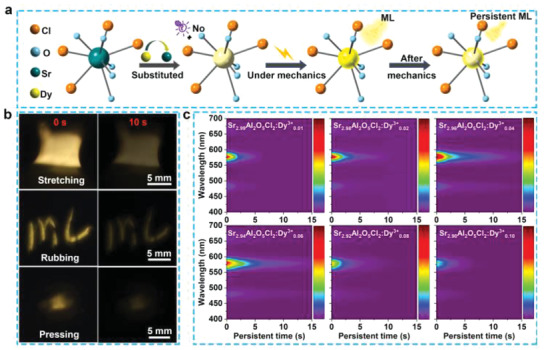
Persistent mechanoluminescence properties of SAOCD/PDMS composite elastomers. a) Schematic of the persistent ML in the structure of SAOCD. b) ML photos of the SAOCD/PDMS composite elastomers after the stimuli of stretching, rubbing and pressing for 0 and 10 s (effective sample size: 25 mm × 10 mm × 1 mm). c) Persistent ML mappings of the SAOCD/PDMS composite elastomers under various doping concentrations of Dy^3+^ ions (stretching mode, stretching frequency: 4 Hz, stretching strain: 100%).

In addition to the ML under mechanics, the SAOCD/PDMS composite elastomer can also exhibit ML afterglow (persistent ML) after the mechanics stimuli, i.e., the ML after removing mechanics could last for ≈15 s observed by naked eyes in dark environment. **Figure** **3**a,b illustrates the entire ML and persistent ML processes as well as their optical photos. The decay curve with more photos for the persistent ML of SAOCD/PDMS after stretching stimulus is presented in Figure S6 (Supporting Information). Figure 3c shows the persistent ML mappings of SAOCD/PDMS by varying the Dy^3+^ contents after stretching stimulus (stretching frequency: 4 Hz, stretching strain: 100%). It is found that the persistent ML of SAOCD/PDMS is highly dependent on the doping content of Dy^3+^, and the sample with 0.04 Dy^3+^ doping exhibits the highest initial intensity and the longest afterglow time. Previously, researchers can only achieve the persistent ML via the trap engineering in the trap‐controlled ML systems.^[^ ^29^, ^30^, ^31^ ^]^ Namely, the persistent ML can only be obtained by the energy releasing from the deep traps to the shallow ones driven by mechanics stimuli, e.g., Ba_0.5_Sr_0.5_Si_2_O_2_N_2_:Eu^2+^,^[^ ^29^ ^]^ LiGa_5_O_8_^[^ ^30^ ^]^ and Sr_2_P_2_O_7_:Eu,Y.^[^ ^31^ ^]^ However, such kind of persistent ML requires pre‐irradiation to fill energy in traps, which is an irreversible process resulting in unrecoverable persistent ML. For the persistent ML in this work, it requires no any pre‐irradiation. Even though the trapped carriers are fully cleared, the SAOCD/PDMS can still exhibit intense ML and afterglow. It suggests that the as‐obtained persistent ML of SAOCD/PDMS should be the self‐charging one under mechanics stimuli, which is different from the previous reports and shows higher application values.

### 2.4 Mechanoluminescence Mechanisms

**Figure 4:**
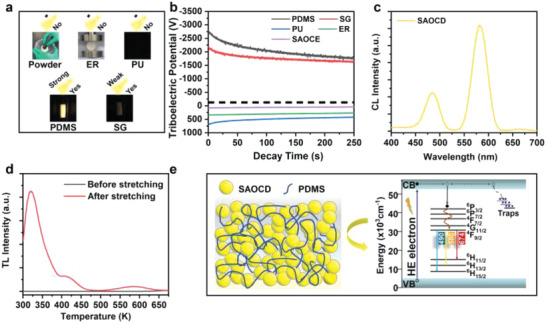
Mechanism of transient and persistent mechanoluminescence of SAOCD/PDMS composite elastomers. a) Illustration of the ML performance of SAOCD in powders and their composites with ER, PU, PDMS, and SG, respectively. b) Triboelectric potential decay rate of various materials when rubbed with SAOCD for 1 min with 60 rpm under 1 N. c) CL spectrum of SAOCD powders. d) TL spectra of SAOCD/PDMS composite elastomers before and after stretching (stretching frequency: 4 Hz, stretching strain: 100%). e) Schematic diagram of the self‐activating ML and self‐charging persistent ML processes of SAOCD/PDMS.

Since the SAOCD/PDMS exhibits unique ML and persistent ML behaviors, it is attractive to further reveal the underlying physical processes. Previously, two types of models in terms of trap‐releasing and piezoelectricity are employed to explain the ML phenomenon.^[^ ^41^, ^42^, ^43^, ^44^ ^]^ However, the as‐observed ML of SAOCD/PDMS in this work is independent from the pre‐stored energy in traps as discussed in Figures 2 and 3. **Figure** **4**a illustrates the matrix effects on the ML performance of SAOCD. It is found that when the SAOCD powders are composited with the PDMS and silicone gel (SG) elastomers, the samples can exhibit ML performance. However, when mechanics stimuli are directly applied on the SAOCD powders (no composite matrix) or their composites with the epoxy resin (ER) and polyurethane (PU), no ML emission can be obtained. The different ML behaviors of SAOCD in various matrices suggest that the interfacial interactions between the SAOCD powders and the polymer chains play critical roles on the ML performance. The non‐ML phenomenon of SAOCD powders and their composites with hard ER under mechanics stimuli further confirms that the ML of the SAOCD should be non‐piezoelectricity‐related, although the doping of Dy^3+^ could produce a certain localized piezoelectricity in the structure. Therefore, the conventional trap‐ or piezoelectricity‐involved mechanisms cannot be employed to explain the ML in this work. Recently, a novel physical model in terms of the interfacial triboelectrification‐induced electron bombardment was proposed by our group to explain the unusual self‐activating ML.^[^ ^32^ ^]^ This model should be established on the following four aspects: i) the ML is non‐piezoelectricity‐related; ii) the ML is independent of traps; iii) the material can exhibit ML only when it is composited with the matrices that can accept electrons under triboelectrification; iv) the ML powders have radiative pathways under the bombardment of high energy (HE) electrons. For the ML of SAOCD, it is non‐piezoelectricity‐related (Figure 4a) and independent of traps (Figure 2f). It can only exhibit ML when compositing with SG and PDMS which could accept electrons under triboelectrification (Figure 4b and Figure S7, Supporting Information). The SAOCD also show obvious cathodoluminescence (CL) (Figure 4c), suggesting that there are effective radiative pathways under the bombardment of high energy electrons. As a result, the ML of SAOCD in SG or PDMS fulfills the interfacial triboelectrification‐induced electron bombardment model, as illustrated in Figure 4e. First, interfacial triboelectrification between the SAOCD particles and the SG or PDMS polymer chains is produced under mechanics stimuli. Then, the electrons are accepted by SG or PDMS, and the interfacial triboelectric field is formed. Under the interfacial triboelectric field, the accepted electrons in SG or PDMS could be accelerated to bombard SAOCD, leading to the electron excitation from valence band (VB) to conduction band (CB). The excited electrons in CB then transfer to the energy levels of Dy^3+^, and finally generate ML emissions after the recombination with holes.

To further understand the persistent ML performance, thermoluminescence (TL) spectra of SAOCD/PDMS before and after stretching stimulus were measured. As shown in Figure 4d, because no pre‐irradiation was applied to pre‐store the energy in traps, the SAOCD/PDMS sample exhibits no TL signal before stretching. However, after the stretching stimulus on the SAOCD/PDMS elastomer (stretching frequency: 4 Hz, stretching strain: 100%), prominent TL signals from 300 to 650 K were obtained. It fully demonstrates that the employed mechanics actions during ML could self‐charge energy in the structure. As suggested in the self‐activating ML processes, the self‐charging process should be the electron transfers from CB to traps based on the interfacial triboelectrification‐induced electron bombardment model, as illustrated in Figure 4e. The self‐filled energy in the shallow trap (≈325 K in the TL spectrum) could spontaneously transfer to the energy levels of Dy^3+^ under the thermal activation of room temperature, and hence the persistent ML was produced. Compared to the conventional persistent ML that requires pre‐irradiation to pre‐store energy in the traps, the developed self‐charging one in SAOCD/PDMS can exhibit persistent ML directly under mechanics stimuli with no need for pre‐irradiation. Therefore, it is more appropriate for the practical application scenarios in terms of the mechanics‐induced lighting, displaying and imaging. As representatives, we have fabricated more patterns (the car and the flower) by the SAOCD/PDMS. As shown in Figure S8 (Supporting Information), these patterns could be efficiently illuminated by the stretching stimulus without any pre‐treatment, after which the displaying could last for about 15 s.

### 2.5 Mechanics Storage and Visualized Reading

**Figure 5:**
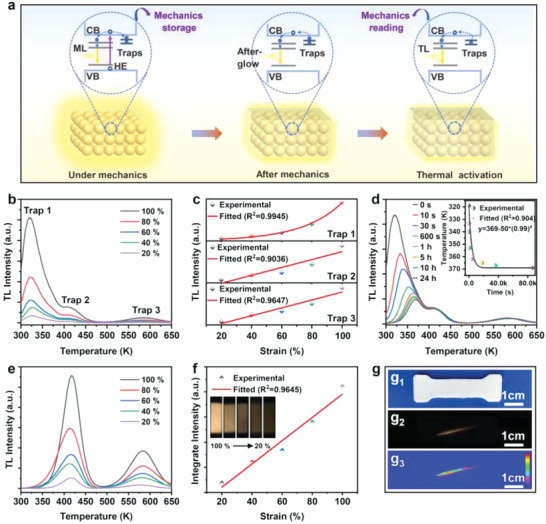
Mechanoluminescence for mechanics storage and reading. a) Schematic diagram of the mechanics storage and its visualized reading. b) TL spectra of SAOCD/PDMS composite elastomers and c) concentration variations of trapped carriers after different stretching strains. d) TL spectra of SAOCD/PDMS after stretching stimulus and standing for different time (stretching frequency: 4 Hz, stretching strain: 100%), the insert shows the corresponding TL peak variations. e) TL spectra and f) TL intensity variation of SAOCD/PDMS after different stretching strains and heat treatment at 373 K for 10 min. g) Optical photos for g_1_) the mechanics storage and g_2_,g_3_) visualized reading (effective sample size: 25 mm × 10 mm × 1 mm).

During the physical processes of the self‐charging persistent ML (Figure 4e), the applied mechanical energy on the SAOCD/PDMS elastomer could arouse carriers to be stored in the structural traps, which could be easily released to emit light by thermal or photon stimulation.^[^ ^45^, ^46^ ^]^ These unique physical processes of the persistent ML of SAOCD/PDMS inspire us an effective strategy to achieve the mechanics storage and visualized reading. As illustrated in **Figure** **5**a, when mechanics are applied on SAOCD/PDMS, distributed ML signals could be exhibited to instantaneously and visually display the applied mechanics. In the meantime, the applied mechanics information is stored in SAOCD/PDMS in the form of trapped carriers. After a period, the stored mechanics information could be visually read out in the form of light by releasing the trapped carriers under thermal activation. To specifically show the mechanics storage and reading activities, the SAOCD/PDMS samples that were stimulated by different stretching strains were investigated. As presented in Figure 5b, the stretching stimuli could fill energy in both shallow and deep traps, and the larger stretching strain corresponds to higher TL intensity. Figure 5c exhibits the detailed relationships between the carrier densities of traps 1–3 and the applied stretching strains. It shows good one to one correspondence, suggesting that the self‐charging carriers in the traps could be employed to achieve the mechanics storage. Because the shallow trap (trap 1) could be thermally activated by room temperature to generate the persistent ML, the mechanics‐charged carriers in trap 1 is gradually decreased with increasing the standing time from 0 to 24 h, as shown in Figure 5d. Meanwhile, the mechanics‐charged carriers in deep traps (trap 2 and trap 3) could be stably existed. Therefore, the applied mechanics strength as well as the occurrence time of mechanics could be simultaneously read out in SAOCD/PDMS by separately utilizing the mechanics‐charged carriers in shallow and deep traps. Namely, we can directly obtain the occurrence time of mechanics from the TL1 intensity or peak location (spontaneously carriers releasing from shallow traps), and further read out the applied mechanics strength by releasing the carriers in deep traps. It should be noted that when reading out the applied mechanics strength, it is necessary to avoid the interference of the shallow traps that dynamically changes. Here, we set pre‐heat‐treatment conditions (373 K for 10 min summarized from Figure S9, Supporting Information) to fully release the carriers in shallow traps, and the stable one to one correspondence between the carrier density of deep traps and the stretching strain is obtained as shown in Figure 5e,f. Based on this relationship, accurate mechanics strength, such as stretching strain and stress, can be easily read out.

To visually present the mechanics storage and reading activities of SAOCD/PDMS, we take the optical photos for each step as shown in Figure 5g. First, mechanics rubbing was applied on the SAOCD/PDMS elastomer. Under daylight, no any change or information can be found (g_1_ in Figure 5g). However, when we placed the SAOCD/PDMS under dark environment and further stimulated it by heating at 673 K, the stored mechanics information could be visually observed by naked eyes (g_2_ in Figure 5g). By further analyzing the luminescence intensity and its distribution (g_3_ in Figure 5g), the applied strength of mechanics rubbing as well as its spatial distribution can be easily read out.

## 3 Conclusion

In summary, the Dy^3+^‐doped Sr_3_Al_2_O_5_Cl_2_ powders were synthesized, and their ML properties were investigated by compositing with PDMS. Different from the conventional ML materials, the Sr_3_Al_2_O_5_Cl_2_:Dy^3+^/PDMS composites exhibited intense and self‐activating ML with no need for any pre‐irradiation. The self‐activated energy by mechanics during ML could be further transferred to the intrinsic traps, and therefore the attractive ML performance in terms of self‐charging persistent ML was created. In addition to facilitating the practical applications in mechanics displaying and visualization, the unique self‐charging processes further endowed the SAOCD/PDMS composites with mechanics storage and visualized reading activities, showing broad application prospects in the fields of mechanical engineering, bioengineering, and artificial intelligence.

## 4 Experimental Section

### Synthesis of SAOCD Powders

A series of Sr_3‐_ *_x_* Al_2_O_5_Cl_2_:*x*Dy (*x* = 0.01, 0.02, 0.04, 0.06, 0.08, and 0.10) powders were synthesized by a high‐temperature solid‐state method. First, stoichiometric amounts of SrCl_2_.6H_2_O (AR), SrCO_3_ (AR), Al_2_O_3_ (AR), and Dy_2_O_3_ (99.99%) raw materials were weighed and thoroughly ground in an agate mortar. Then, the mixture was pre‐sintered at 800 °C for 2 h under air in a muffle furnace, and sintered at 1200 °C for 4 h under the atmosphere of 90% N_2_ and 10% H_2_ in a tube furnace. After cooling to room temperature, the SAOCD powders were obtained.

### Fabrication of SAOCD‐Based Composites

To quantitatively analyze the ML properties, SAOCD powders were embedded into transparent PDMS (Sylgard 184, Dow Corning) with a powder‐to‐polymer mass ratio of 1:1. First, PDMS base resin (2 g) and curing agent (0.2 g) with a mass ratio of 10:1 were mixed in a petri dish (diameter: 30 mm). Then, 2 g of SAOCD powders were dispersed into the above PDMS precursor by mechanical stirring for 10 min to form a homogeneous paste. After that, the mixture was poured into a dumbbell‐shaped mold (25, 10, and 1 mm in effective length, width, and thickness, respectively) and removed the gas under negative pressure (room temperature; −80 kPa) for 10 min. After curing at 80 °C for 30 min, standard test samples of SAOCD/PDMS composite elastomers were obtained.

The preparation processes of SAOCD/PU, SAOCD/SG and SAOCD/ER were similar to the fabrication of SAOCD/PDMS, and the curing conditions were adjusted to 60 °C for 120 min. The ratio of base resin: curing agent: SAOCD for SAOCD/PU, SAOCD/SG and SAOCD/ER were 1:1:2, 1:1:2, and 3:1:1, respectively.

### Characterizations

Powder XRD patterns of the as‐synthesized SAOCD were carried out on an XRD‐6100 (Shimadzu. Japan) X‐ray diffractometer with CuK*α* radiation (*λ* = 1.54178 Å). The morphology and element distribution of the samples were obtained by SEM (FEI Quanta 650 FEG) with energy dispersive X‐ray spectroscope fittings (EDAX ELEMENT). The TL curves were measured using a microcomputer thermoluminescence spectrometer (FJ427A1, Beijing Nuclear Instrument Factory) at a heating rate of 1 Κ s^−1^. ML signals were collected in situ from a homemade tensile testing machine using a high‐throughput optical fiber. The ML spectra were recorded by a fluorescence spectrophotometer (Omni‐*λ*300i, Zolix Instruments Co., Ltd.) equipped with a CCD camera (iVac‐316, Edmund Optics Ltd.). The stress‐strain curve of SAOCD/PDMS elastomer was applied by a universal testing machine (Shimadzu AGS‐X‐500 N). The triboelectric potential was collected by using the electrostatic measuring probe (SK050, KEYENCE (Japan) Co., Ltd.) at a distance of 10 mm. The CL spectrum of SAOCD was detected on the modified Mp‐Micro‐S instrument attached to the SEM. High‐temperature environments were provided by the heating table (JF‐956A). All optical photos were taken by a digital camera (Canon EOS 77D).

### Statistical Analysis

The horizontal comparison of the ML properties in Figures 2d,f and 3c was tested at the same stretching conditions (stretching frequency: 4 Hz, stretching strain: 100%). The error analysis in Figure 2e,f was obtained from three parallel tests. The curve fittings in Figure 5c,d,f were performed by using the Origin software (version 2021).

## Conflict of Interest

The authors declare no conflict of interest.

## Supporting information

Supporting Information

## Data Availability

The data that support the findings of this study are available from the corresponding author upon reasonable request.
