# Supplementary material for: Self‐Charging Persistent Mechanoluminescence with Mechanics Storage and Visualization Activities
Source: Adv Sci (Weinh). 2022 Aug 17;9(28):2203249. doi: 10.1002/advs.202203249 (PMC9534939; doi:10.1002/advs.202203249)
Supplement: Supplementary file 1 — Supporting Information [file ADVS-9-2203249-s001.pdf]

ADVANCED SCIENCE
Open Access

Supporting Information

for *Adv. Sci.*, DOI 10.1002/advs.202203249

Self-Charging Persistent Mechanoluminescence with Mechanics Storage and Visualization Activities

*Yongqing Bai, Xiuping Guo, Birong Tian, Yongmin Liang, Dengfeng Peng and Zhaofeng Wang**

Supporting Information

Self-charging persistent mechanoluminescence with mechanics storage and visualization activities

*Yongqing Bai, Xiuping Guo, Birong Tian, Yongmin Liang, Dengfeng Peng, and Zhaofeng Wang**

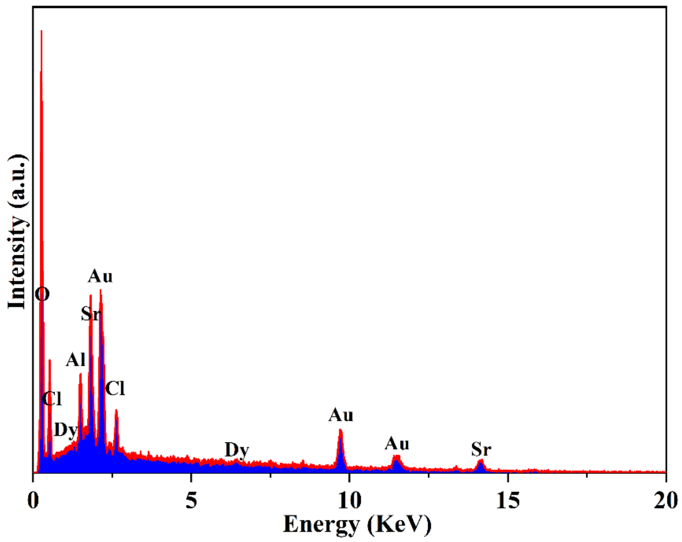

Figure S1 EDS profile of the as-synthesized SAOCD powders.

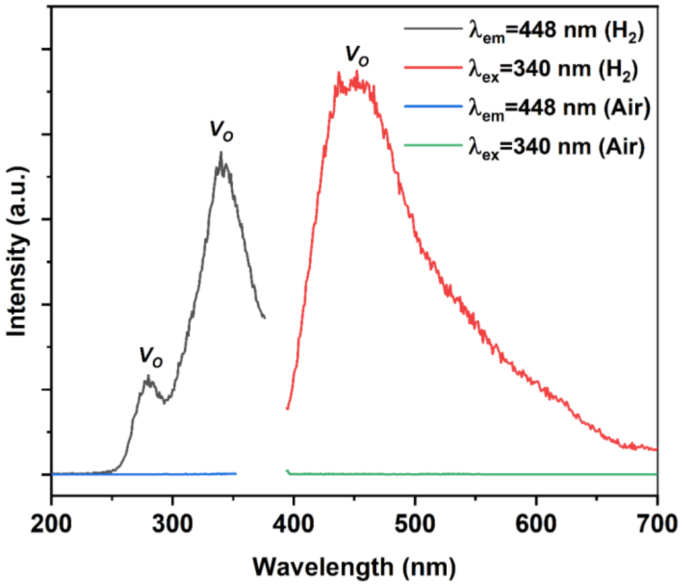

Figure S2 PL and PLE spectra of the $Sr_3Al_2O_5Cl_2$ samples synthesized under air and reducing atmosphere. The reduction atmosphere is beneficial to the generation of oxygen vacancies in the structure of $Sr_3Al_2O_5Cl_2$, which has been widely reported in previous work. Therefore, the as-observed PL and PLE peaks of the sample synthesized under reduction atmosphere are confirmed to be aroused by the intrinsic oxygen vacancies.

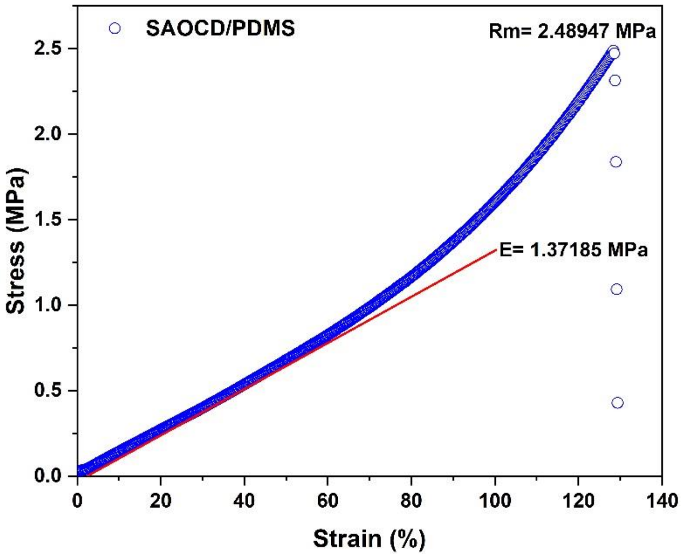

Figure S3 Stress-strain curve of the SAOCD/PDMS composite elastomer tested on a universal testing machine.

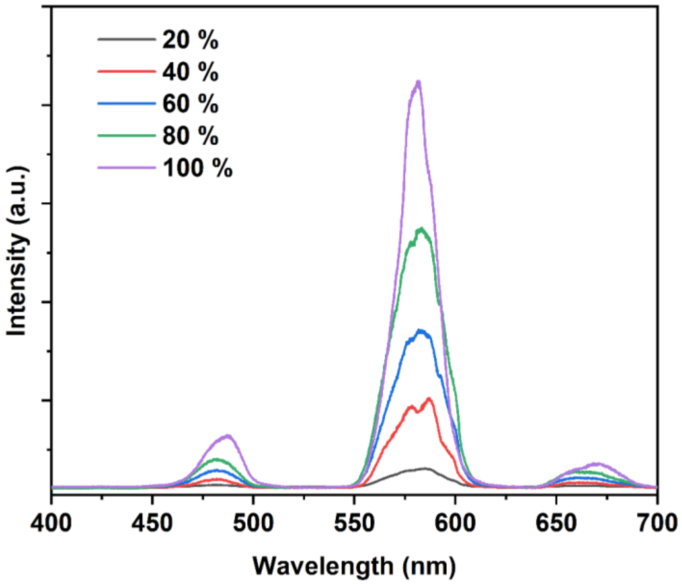

Figure S4 Strain-dependent ML spectra of $Sr_{2.96}Al_2O_5Cl_2$:0.04Dy in PDMS under stretching mode with the frequency of 4 Hz.

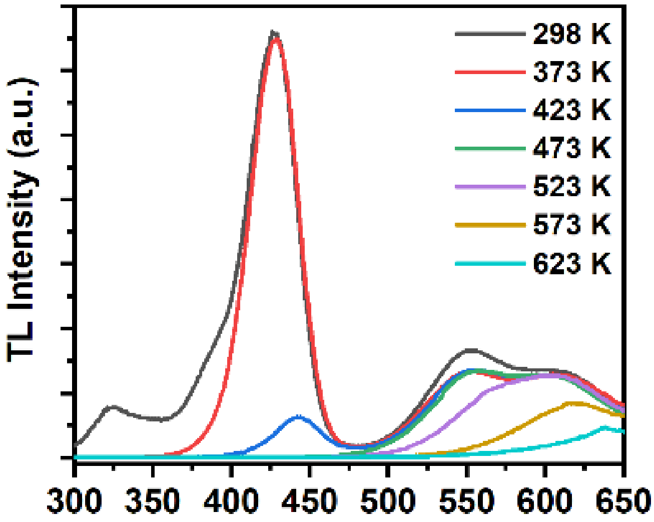

Figure S5 TL spectra of SAOCD after heat-treatment at various temperature for 10 min. The samples were pre-irradiated by a UV lamp for 5 min before TL test.

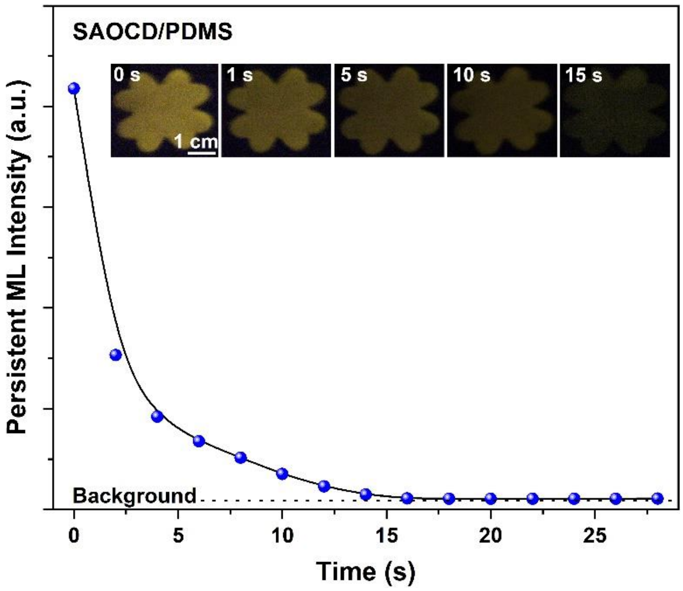

Figure S6 Decay curve for the persistent ML of SAOCD/PDMS after stretching stimulus, the insets show the persistent ML photos at 0, 1, 5, 10 and 15 s.

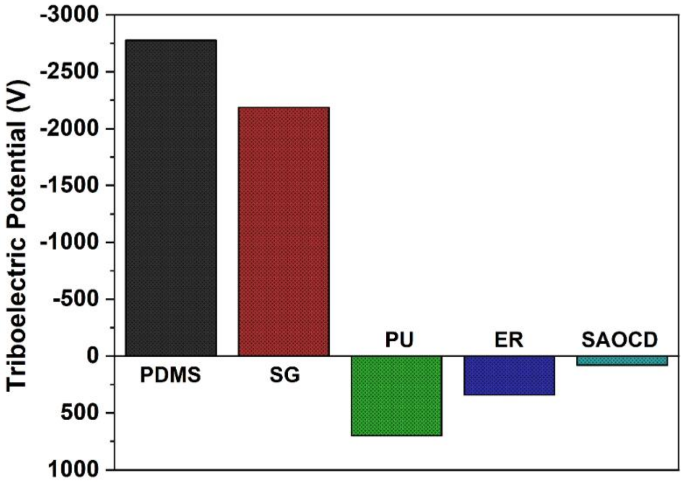

Figure S7 Triboelectric potential of PDMS, SG, PU, ER and SAOCD when rubbing with the SAOCD for 1 min with 60 rpm under 1N.

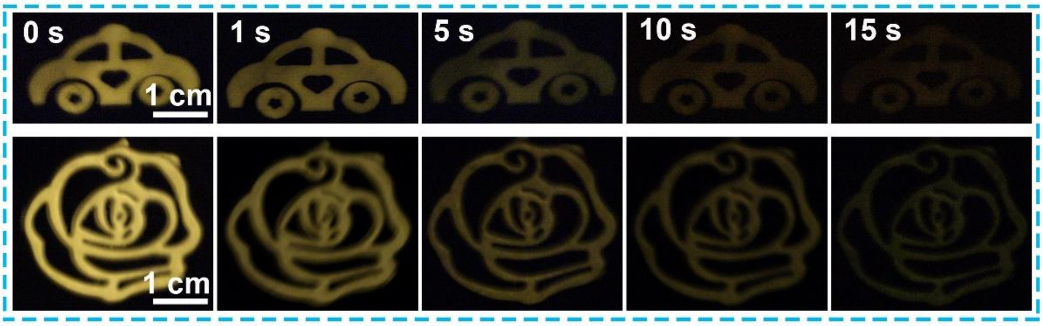

Figure S8 More patterns fabricated from the as-developed SAOCD/PDMS composites for the persistent displaying activities after mechanics stimuli.

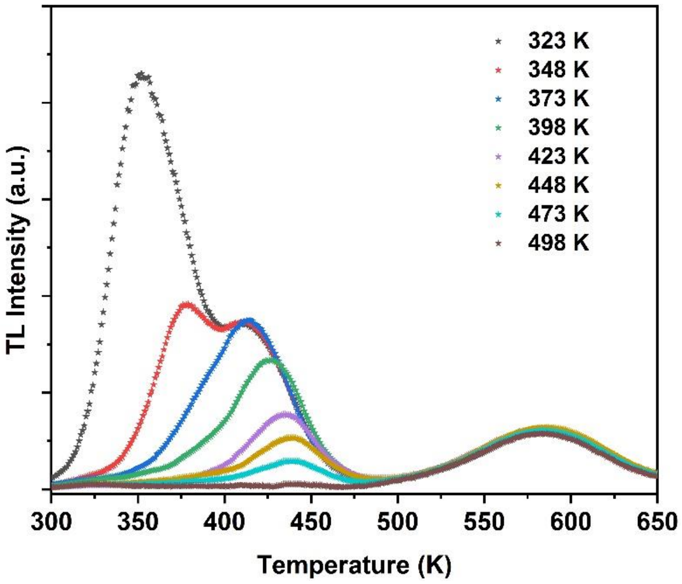

Figure S9 TL spectra of SAOCD/PDMS after heat-treatment at various temperature for 10 min. The samples were pre-filled energy by stretching before TL test.
